# The use of radiochromic EBT2 film for the quality assurance and dosimetric verification of 3D conformal radiotherapy using Microtek ScanMaker 9800XL flatbed scanner

**DOI:** 10.1120/jacmp.v14i4.4182

**Published:** 2013-07-08

**Authors:** GS Sim, JHD Wong, KH Ng

**Affiliations:** ^1^ Department of Biomedical Imaging Faculty of Medicine, University of Malaya Kuala Lumpur Malaysia; ^2^ Pantai Hospital Ayer Keroh No. 2418‐1, Km 8 Lebuh Ayer Keroh Melaka Malaysia; ^3^ University of Malaya Research Imaging Centre (UMRIC) University of Malaya Kuala Lumpur Malaysia

**Keywords:** anthropomorphic phantom, dosimetry, radiochromic EBT2 film, treatment planning system, Microtek ScanMaker 9800XL

## Abstract

Radiochromic and radiographic films are widely used for radiation dosimetry due to the advantage of high spatial resolution and two‐dimensional dose measurement. Different types of scanners, including various models of flatbed scanners, have been used as part of the dosimetry readout procedure. This paper focuses on the characterization of the EBT2 film response in combination with a Microtek ScanMaker 9800XL scanner and the subsequent use in the dosimetric verification of a 3D conformal radiotherapy treatment. The film reproducibility and scanner uniformity of the Microtek ScanMaker 9800XL was studied. A three‐field 3D conformal radiotherapy treatment was planned on an anthropomorphic phantom and EBT2 film measurements were carried out to verify the treatment. The interfilm reproducibility was found to be 0.25%. Over a period of three months, the films darkened by 1%. The scanner reproducibility was ± 2% and a nonuniformity was ±1.9% along the direction perpendicular to the scan direction. EBT2 measurements showed an underdose of 6.2% at high‐dose region compared to TPS predicted dose. This may be due to the inability of the treatment planning system to predict the correct dose distribution in the presence of tissue inhomogeneities and the uncertainty of the scanner reproducibility and uniformity. The use of EBT2 film in conjunction with the axial CT image of the anthropomorphic phantom allows the evaluation of the anatomical location of dose discrepancies between the EBT2 measured dose distribution and TPS predicted dose distribution.

PACS number: 87.55.Qr

## I. INTRODUCTION

Conventional detectors such as ionization chambers, semiconductor detectors, and thermoluminescent detectors (TLD) have been used for radiation therapy dosimetry. These detectors are often used to validate isodose curves and depth‐dose distribution. One of the limitations of these single volume dosimeters is that they do not provide two‐dimensional dose information. For this type of measurement, film or 2D ion chamber/diode arrays may be the better choice. Two‐dimensional ion chamber/diode arrays are also limited in spatial resolution due to the ion chamber size and spacing between neighboring ion chambers/diodes; thus they are not suitable for measurements with high spatial resolution. Radiographic film is another type of dosimeter, but it has large energy dependence due to the high‐Z material in the film. This results in the film over‐responding at low energies. In addition, it is very sensitive to light and requires wet chemical processing.

In recent years, radiochromic EBT/EBT2 film has gradually gained widespread use in radiation dosimetry. Radiochromic film is a self‐developing film, hence does not require the use of conventional chemical processing. This film is less sensitive to room light, resulting in easy handling. EBT2 films have very low energy dependence, with a 10% difference between 6 MV and keV photons.^(1)^ By contrast, variations of the response of radiographic film exceed one order of magnitude over the same range.^(2)^

Over the years, many different scanners and scanner models have been used for film dosimetry ranging from professional film scanners, such as VIDAR VXR Dosimetry Pro Advantage scanner to commercially available flatbed scanners.^(3)^ The use of flatbed scanners are appealing due to their lower cost. The response and characteristics of each type of scanner have been studied by various groups.^3^, ^4^, ^5^ This is necessary so as to optimize the use of the respective scanner and to minimize the uncertainty in the measured dose due to scanner‐related parameters. Epson Expression 10000XL scanner is widely used as the choice flatbed scanner for EBT2 dosimetry, perhaps due to the recommendation by the EBT2 manufacturer. However, at our center, the only available flatbed scanner is a Microtek ScanMaker 9800XL scanner. The aim of this study was to use resources that are readily available at our center for radiochromic film dosimetry, despite not being the currently recommended scanner. This paper focuses on the characteristics of the EBT2 film response in combination with an A3‐sized flatbed scanner, Microtek ScanMaker 9800XL scanner, and to study the use of EBT2 radiochromic film in the dosimetric verification of a 3D conformal radiotherapy treatment. To the best of the authors’ knowledge, the characteristics of the Microtek ScanMaker 9800XL scanner have not been reported with the use of EBT2 film in radiation therapy dosimetry.

## II. MATERIALS AND METHODS

### A. EBT2 film calibration, scanning and analysis protocol

GAFCHROMIC film EBT2 (ISP, Wayne, NJ) (Lot no.: A06271101) was used in this work. The films were handled with care to avoid fingerprints and were prepared on a clean surface. The orientation of the film was marked as soon as it was taken out from the box to minimize inaccuracies in measured optical density and thus measured dose due to orientation effects.^(6)^

A total of 24 pieces of cut films were irradiated with doses ranging from 20 to 300 cGy using a Varian 2100 CD linear accelerator (Varian Medical Systems, Palo Alto, CA). Three pieces of cut film were irradiated at the same time for each dose range. The calibration films were positioned at 1.5 cm depth in solid water and irradiated using a 6 MV photon beam. The field size was set to $10\times 10{\text{cm}}^{2}$ and source‐to‐surface distance (SSD) of 100 cm. The linac output was calibrated following the IAEA TRS398 protocol using an ion chamber with Secondary Standard Dosimetry Laboratory calibration.

The calibration curve was tested for its accuracy using films irradiated with three different setups: i) field size of $15\times 15\;{\text{cm}}^{2}$, depth of 3 cm, 155 MU; ii) $10\times 10\;{\text{cm}}^{2}$, depth of 5 cm, 200 MU; iii) $12\times 12\;{\text{cm}}^{2}$, depth of 4 cm, 275 MU. Three pieces of film were used for each setup.

**Figure 1:**
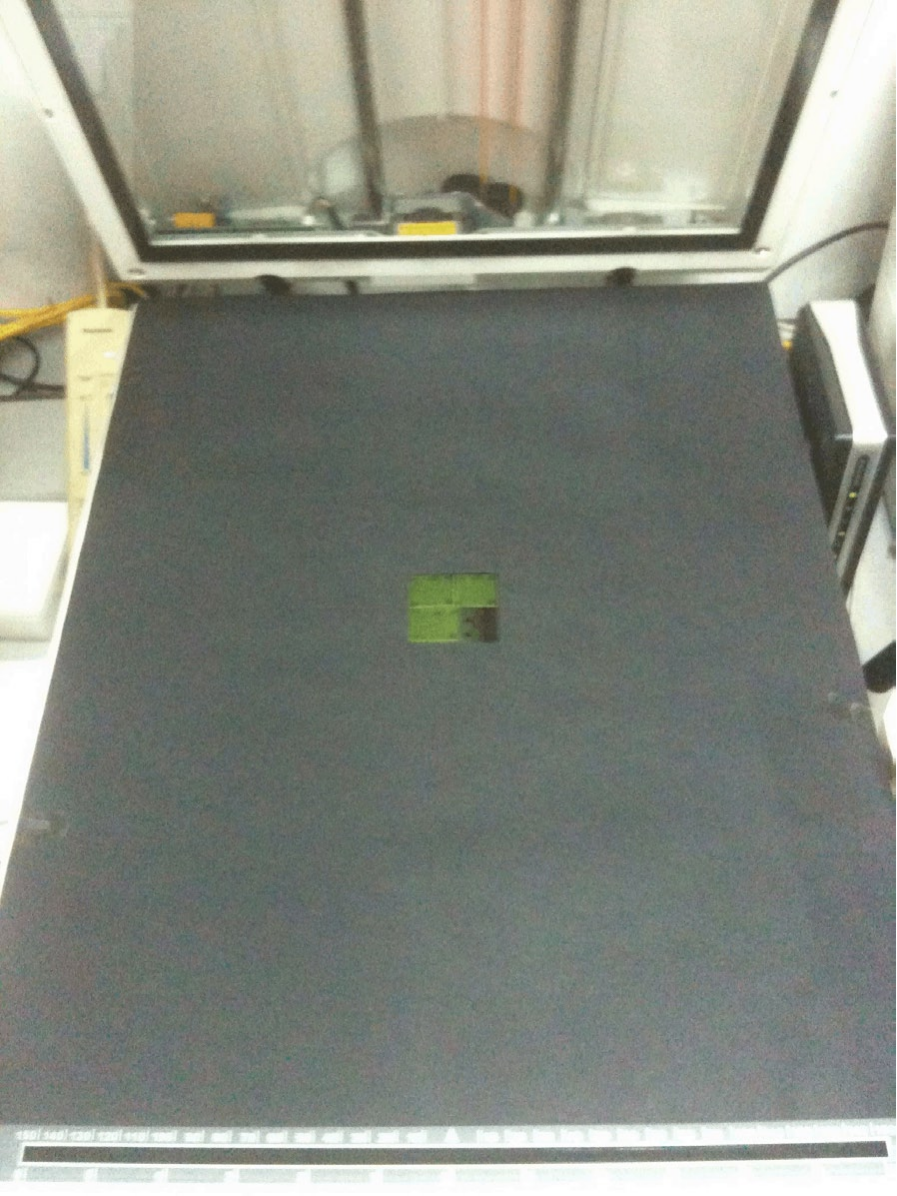
A custom‐made film frame was placed on the scanner bed to ensure reproducible positioning of the calibration films at the center of the scanner bed.

The EBT2 films were scanned using a Microtek ScanMaker 9800XL (Microtek Inc. Santa Fe Spring, CA) 24 hours after irradiation. This is to allow for maximum postirradiation coloration.^(7)^ To maintain the scanning position, a film frame was made and was placed on top of the scanner bed (Fig. 1). Scanning orientation was kept consistent for all films. This is because EBT2 film exhibits a different response in portrait orientation compared to the response in landscape orientation of 7%–9%.^(8)^

All films were scanned in three colors (48 bit RGB) at a scanning resolution of 75 dpi in transmission mode. The scanning software used was Microtek ScanWizard Pro (Microtek Inc.). The scanning direction was parallel to the long axis of the scanner bed size of 30.5cm×43.2cm. Care was taken to ensure that all films were consistently scanned with the same face towards the light source of the scanner.^(9)^ The films were scanned six times, but only the last three scans were kept for analysis and saved as tagged image file format (.tiff files).

The image analysis software used for the film analysis is ImageJ 1.43u (National Institute of Health, Bethseda, MD). Data from the red channel were extracted using a region of interest (ROI) of $1.25\times 1.25{\text{cm}}^{2}$. Judicious selection of the ROI allowed for avoidance of possible film artifacts due to the cutting of the film at the film edge, hence reducing the noise and uncertainty of inter‐film readings. Mean pixel values were obtained and optical density (OD) was calculated using Eq. (1):

(1) $$OD={\text{log}}_{10}\frac{PV_{unirradiated}}{PV_{afterirradiation}}$$

where ${\text{PV}}_{\text{unirradiated}}=$ mean pixel value of unirradiated films, and ${\text{PV}}_{\text{afterirradiation}}=$ mean pixel value of film after irradiation.

### B. Scanner reproducibility measurements

The scanner reproducibility was studied using an optical density step‐wedge radiographic film. The film was scanned in three sessions at two‐hour intervals. In each session, the film was scanned rapidly for ten times consecutively. The same procedure was repeated the next day.

Scanner warm‐up characteristics, and intersession and intrasession variability were investigated. The optical density was obtained as a step‐wedge profile, averaged over a width of 1 cm.

### C. Film reproducibility measurements

The films were scanned on different days to test the film's reproducibility. A set of films comprising of film strips irradiated with selected known doses were scanned seven times over a period of three months. Throughout this time, the set of films were stored in a cool, dark place and was only taken out for periodic rescans. It has been postulated that the EBT2 film may darken due to two factors; (i) natural darkening (possible effects of time, temperature, and humidity), and (ii) multiple scanning of the same film (ultraviolet exposure from the scanner light). To isolate and identify the two effects, another set of films were exposed to similar dose levels and irradiated on the same date, but only subjected to two scans over the same period of time — once 24 hours after exposure and once after three months. The mean PV for these films was obtained and normalized to the mean PV of the first scan. The darkening of the films in the two sets was tracked over a period of three months. The degree of darkening was compared to the films that were subjected to multiple irradiation over the period of 95 days.

### D. Scanner uniformity measurements

Radiochromic film dosimetry is often limited by scanning parameters. When scanning the radiochromic film with a flatbed scanner, the film response over the scan area is not uniform due to different light scattering conditions from the scanner light.^(10)^ The uniformity effect in the scanner was tested for the direction parallel and perpendicular to the scan direction.

For each of the scanner directions, the scanner uniformity was studied by scanning a whole piece of unirradiated EBT2 film in two different orientations, (i) portrait and (ii) landscape. The landscape orientation is the placement of the short edge of the film parallel to the scan direction.

### E. Dosimetric verification of a 3D conformal radiotherapy treatment plan

The EBT2 film was used for the dosimetric verification of a three‐field 3D conformal radiotherapy (3D CRT) lung treatment plan. A one‐year‐old pediatric anthropomorphic phantom (CIRS, VA) was used in this study. The phantom was scanned in a Philips Brilliance Big Bore computed tomography (CT) simulator (Royal Philips Electronics, Amsterdam, The Netherlands). The images from the CT simulator were then exported to the Eclipse treatment planning system (TPS), version 8.9 (Varian Medical Systems) for external beam planning.

**Figure 2:**
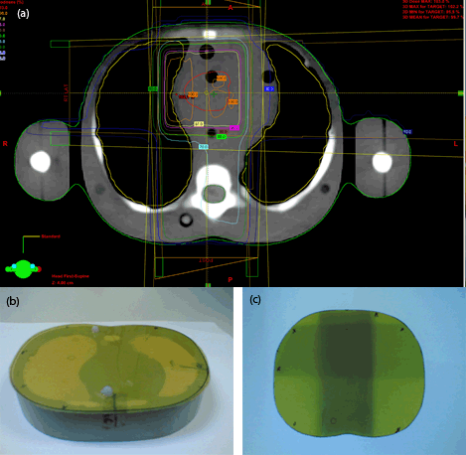
(a) Eclipse treatment plan showing the delineated PTV and organ at risks, (b) EBT2 film placed on the anthropomorphic phantom in the transverse plane and (c) irradiated EBT2 film.

The Eclipse TPS was used for delineation of planning target volume, lung, and spinal cord. The anthropomorphic phantom was irradiated according to the treatment plan, with the EBT2 film sandwiched between the phantom slabs in the transverse position (Fig. 2). The film was cut according to the contour of the phantom body. The measurements were repeated twice. After the exposure, the films were digitized and analyzed using ImageJ (as described in Material and Methods section A above). The dose profile obtained from EBT2 measurements was plotted and comparison was made with the dose profile from the TPS at the same region of measurement.

Two‐dimensional comparison was also made between the EBT2 measured and TPS predicted planar dose distribution using an in‐house code written in MATLAB 2008b (The MathWorks Inc., Natick, MA). The 2D dose distribution was compared using gamma index.^(11)^ A gamma criteria of 7% dose difference and 7 mm distance‐to‐agreement (DTA) was employed to compare the two dose distributions.

## III. RESULTS & DISCUSSION

### A. Calibration curve

**Figure 3:**
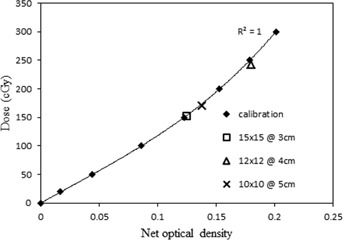
Film calibration curve.

EBT2 films were calibrated at a known dose ranging from 20 to 300 cGy and the PV obtained from ImageJ was converted to OD using Eq. (1). The dose was plotted as a function of net optical density (Fig. 3). The average coefficient of variations of three pieces of films, irradiated within the same setup, was 0.25%. The dose response data were fitted with a third order polynomial function ($r_{2}=1.0$).

To test the accuracy of the calibration curve, the films with three different setups were overlayed onto the calibration curve. The measured dose from EBT2 was compared with calculated dose using Eq. (2) and tabulated in Table 1. The maximum difference between the measured and calculated dose was found to be 3.9%.

(2) $$\text{Calculated dose}=\frac{\text{MU}\times (\text{depth dose}/100)}{\text{FSCF}}$$

where *FSCF* is the field size correction factor obtained from the physics beam data, and *MU* is the monitor unit.

**Table 1:** Test film results and comparison between measured and calculated dose

*Field Size (cm* ^*2*^ *)*	*Depth (cm)*	*MU*	*% Depth Dose*	*FSCF* ^a^	*Calculated Dose (cGy)*	*Measured Dose (cGy)*	*Difference (%)*
15×15	3	155	94.5	0.967	151.40	150.70	0.5
10×10	5	200	85.9	1.000	171.90	170.10	1.0
12×12	4	275	90.4	0.985	252.39	242.59	3.9

a FSCF at our center is taken as the ratio of the output of the reference field size (10×10) divided by the output of the field size of interest.

### B. Scanner reproducibility

The Microtek ScanMaker 9800XL flatbed scanner showed an intrasession mean coefficient of variation of 1.6±0.3%(1SD), while the intersession coefficient of variation was 1.9%. The scanner reproducibility is poorer compared to an Epson Expression 10000 XL scanner.^(4)^ The scanner does not appear to have a warm‐up behavior when the film was scanned consecutively for ten times.

### C. Film reproducibility

**Figure 4:**
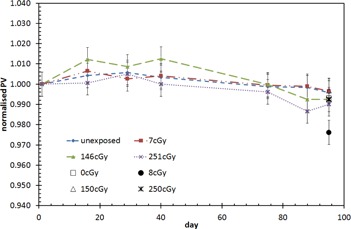
Comparison of film response over time. Dotted and dashed lines are plotted for visual guidance. Error bars represent 1 SD for the mean of four films.

Figure 4 shows the results for film reproducibility. It can be seen that the film response changes over time, with the average darkening of 0.6±0.2%(1SD) after 95 days. The variation of the PVs was within the measurement uncertainties (shown as the error bars in Fig. 4) with the first 40 days. The normalized PV of the films that were subjected to multiple scanning (seven times) appears to be similar to those that were scanned twice, with the exception of the film that was irradiated with 8 cGy showing a darkening of up to 2.5%. Average darkening was 1.0±0.9%. The PVs appear to show a decreasing trend starting from day 40. However, the magnitude of change in the PV is still within the uncertainty of the scanning reproducibility of 2%.

This study shows that the EBT2 film darkens with time. Hence, it may be prudent to establish a calibration curve every time measurements are performed.^(12)^ However, if this is not possible, perhaps due to limited resources, the use of the calibration curve obtained within a period of three months may be used, taking into account the possible film darkening of up to 1%.

### D. Effects of scanner uniformity

**Figure 5:**
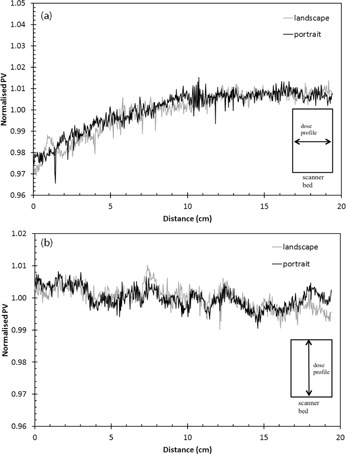
Scanner uniformity in the direction (a) perpendicular and (b) parallel to scan direction.

The uniformity of the scanner was studied by obtaining the profiles in the direction perpendicular and parallel to the scanning direction. The PV was normalized to the mean PV of the respective profile. The profile of normalized PV in the direction perpendicular and parallel to the scan direction was plotted and superimposed with the two different film orientations (Fig. 5).

The profile in the direction perpendicular to the scan direction (Fig. 5(a)) shows a nonuniformity of ±1.9%(2SD). This is due to the nonuniformity of the scanner light source and/or light scattering. A similar study of EBT film conducted by Lynch et al.^(13)^ demonstrated that scanning uniform radiochromic films demonstrated a distinct bowing effect in the direction of the charged coupled device (CCD) array with a nonuniformity of up to 17% using Epson Expression 1680 and 8% in Microtek ScanMaker i900. This effect was also observed by other groups using different flatbed scanners.^3^, ^4^, ^5^, ^14^ It was also noted that the bowing effects increased with films that were irradiated with higher doses.^4^, ^5^, ^13^ Lynch et. al. strongly recommended adherence to a strict scanning protocol that includes maintaining the orientation of films scanned on flatbed scanners and limiting scanning to the central portion of the scanner bed.^(13)^ In the direction parallel to the scan direction (Fig. 5(b)), the nonuniformity was found to be ±0.65%(2SD). Therefore, no correction needs to be made if the dose profile is to be measured in this direction.

To address the issue of scanner‐induced nonuniformity, various groups have proposed different correction methods.^4^, ^5^, ^14^ However, Farreira et al.^(4)^ have also questioned the need for this correction as the introduction of correction factors may also increase the uncertainty of the dosimetry without improving the agreement between the planned and delivered dose distribution. In our work, no correction method was proposed to correct for the scanner nonuniformity because the nonuniformity was ±1% within the scan region that was used. More work may be required to understand the scanner response with respect to films irradiated to different radiation dose levels in order to provide an accurate correction method.

### E. Dosimetric verification of a 3D conformal radiotherapy treatment plan

**Figure 6:**
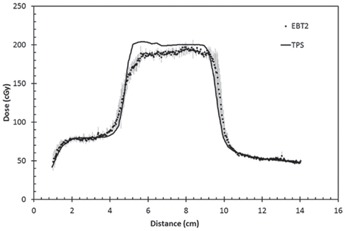
Dose profile comparison of EBT2 and TPS. (Error bars represent 1 SD of the mean of three films.)

A horizontal profile positioned at the central axis of the lateral beam was extracted from the EBT2 film measurement. This was compared with the dose profile at the same position, in the axial plane, obtained from the Eclipse TPS (Fig. 6). The average deviation of the two profiles was 3.5% (1 SD). However, for the region where the TPS predicted dose was >95% of the prescribed dose (2 Gy/fraction), the measured dose appears to underdose by an average of 6.2±3.4%(1SD). This discrepancy between the predicted and measured dose appears to be inconsistent with the recommendation of the International Commission of Radiation Units and Measurements (ICRU) that the absorbed dose to the target volume be delivered with uncertainty less than ±5%.^(15)^ This may be partly due to the uncertainty of the scanner reproducibility and scan bed nonuniformity. However, it is also noted that areas of large discrepancy were located within the lung region and interface of the lung cavities with soft tissue. The quality assurance of treatment planning system for radiotherapy was also reported in detail by the AAPM Task Group 53 and IAEA TRS 430.^16^, ^17^ In these reports, the acceptable criteria for external beam dose calculation in an inhomogeneous phantom is ±7% and 7 mm. The criteria were based on the collective decision of the committee, considering the various uncertainties and limitations of the dose calculation algorithm.

**Figure 7:**
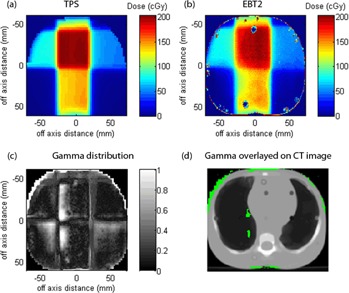
TPS generated dose distribution, (b) EBT2 measurements (average of three measurements) scanned at 75 dpi, (c) gamma distribution (gamma criteria=7%/7mm), and (d) gamma distribution (gamma7%/7mm>1) overlaid on CT image.

The use of film dosimetry allows 2D dose distribution evaluation, as shown in Fig. 7. This figure shows the comparison of the EBT2 film measurement with the TPS‐generated dose distribution. A gamma criteria of 7% dose difference and 7 mm DTA were used for analysis.

Film dosimetry used with anthropomorphic phantom allowed the measurement of the actual beam delivery with respect to realistic human anatomy and tissue inhomogeneities. Overlaying the gamma distribution on the CT axial image also enabled one to determine the anatomical location where disagreement between the planned and measured dose exists, as shown in Fig. 7(d). In this case, it is clear that the majority of the disagreement between the planned and measured dose distribution is located at the edge of the phantom and in the lung region. Gamma failure at the edge of the phantom may be due to the artifacts in the film where the EBT2 film was cut to the size of the phantom and pen markings in defining the film orientation. However, it is interesting to note the disagreement between the planned and measured dose distribution in the lung region. The TPS‐predicted dose distribution showed slightly higher dose delivered to the lung region adjacent to the soft tissue structure. This may indicate the inability of the treatment planning system in predicting the correct dose distribution in the presence of tissue inhomogeneities. The Eclipse TPS at this center uses pencil beam convolution (PBC) algorithm. The inhomogeneity correction used by this algorithm is based on equivalent tissue air ratio (ETAR) method,^18^, ^19^, ^20^ which has been found to have a less accurate dose prediction for air cavities and interfaces.^20^, ^21^, ^22^, ^23^

## IV. CONCLUSIONS

In this work, radiochromic EBT2 films were used in combination with a Microtek ScanMaker 9800XL flatbed scanner. This scanner is readily available and is a cost‐effective scanner solution for radiochromic film dosimetry at our center. This work has characterized this scanner for the use of film dosimetry.

Radiochromic film dosimetry requires a very consistent procedure during calibration and measurement, film digitization, and film evaluation in order to achieve high precision and accuracy in radiotherapy quality assurance and dose verification work. The interfilm reproducibility was 0.25%. Multiple scans over a three‐month period showed a 1% darkening of the films. The scanner reproducibility was ±1.9%. When used for film dosimetry, one should take into account the scanner reproducibility and the uncertainty required for radiation dosimetry. The nonuniformity of the scanner was ±1.9% along the direction perpendicular to the scan direction due to the nonuniformity of the light source and/or light scattering. Scanner nonuniformity was ±0.65% in the direction parallel to the scan direction. Further work is required to study the scanner uniformity with respect to films irradiated to different dose levels before one can consider applying any correction matrix to correct for the scanner bed nonuniformity.

The EBT2 film was used for the dosimetric verification of 3D conformal radiaotherapy treatment on an anthropomorphic phantom. The EBT2 measurement and the TPS‐predicted dose were compared in 1D profile in terms of dose difference and 2D dose distribution using the gamma index. The average deviation of the 1D profiles was 3.5%.

Absorbed dose measured at the interface of lung cavity and soft tissue showed an underdose of 6.2%. This may be due to the inability of the treatment planning system in predicting the correct dose distribution in the presence of tissue inhomogeneities, uncertainty of the scanner reproducibility, and scan bed nonuniformity. The use of EBT2 film in conjunction with the axial CT image of the anthropomorphic phantom allows the evaluation of the location of dose discrepancies between the EBT2‐measured dose distribution and TPS‐predicted dose distribution.

## ACKNOWLEDGMENTS

The authors would like to thank the radiotherapist and medical physicists from the University of Malaya Medical Centre (Mr. Lee Chia Hui, Ms. Munira Rejab, and Ms. Zulaikha Jamalluddin) for their help with the 3D CRT measurements. The authors thank Mr. Jong Wei Loong for his help with scanning some of the films. This work has been supported by the University Malaya grant (PPP: P0079/2012A). The authors would also like to thank Dr. Sou‐Tung Chiu‐Tsao for her valuable comments in the manuscript preparation.
